# T2-hyperintensity in the internal globus pallidus in Machado-Joseph disease

**DOI:** 10.1055/s-0043-1772605

**Published:** 2023-10-13

**Authors:** Alex T. Meira, Jorge E. D. Araruna, Lais Vieira Araujo, Raíssa N. L. F. Leite, Gustavo L. Franklin, Adriana M. T. Nepomuceno, Hélio A. G. Teive

**Affiliations:** 1 Universidade Federal da Paraíba, Departamento de Medicina Interna, Serviço de Neurologia, João Pessoa PB, Brazil.; 2 Nova Diagnóstico por Imagem, João Pessoa PB, Brazil.; 3 Pontifícia Universidade Católica, Departamento de Medicina Interna, Serviço de Neurologia, Curitiba PR, Brazil.; 4 Hospital Universitário Lauro Wanderley, João Pessoa PB, Brazil.; 5 Universidade Federal do Paraná, Hospital de Clínicas, Departamento de Medicina Interna, Serviço de Neurologia, Curitiba PR, Brazil.

**Figure 1:**
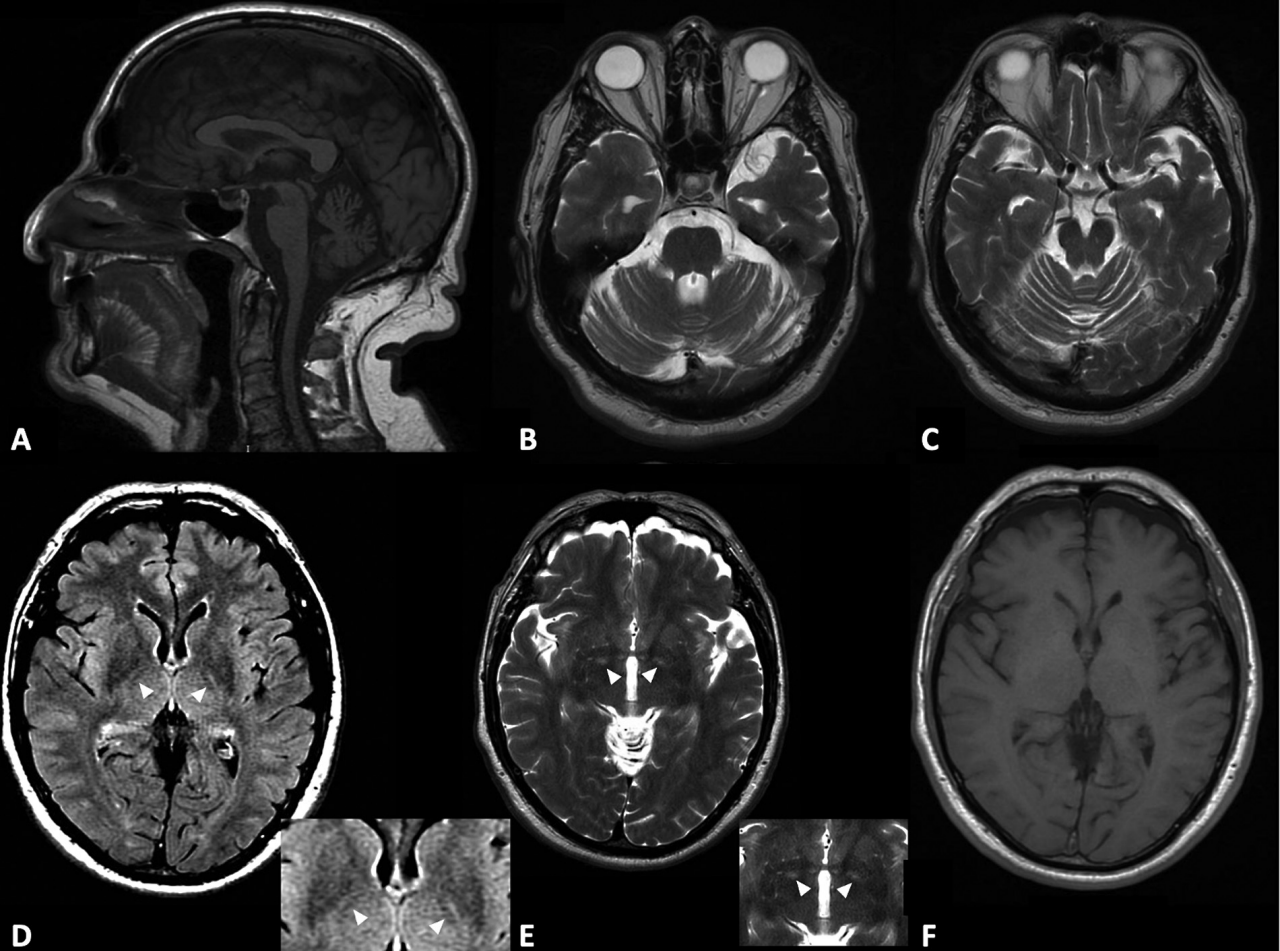
T2 and FLAIR hyperintensity in a patient with Machado-Joseph disease (SCA3). Cerebellar atrophy is shown in sagittal T1 ( **A** ), and axial T2 ( **B** and **C** ). Hyperintensity in the medial aspect of the internal globus pallidus is shown in the FLAIR ( **D** ) and T2 ( **E** ), but in T1 ( **F** ) there is no sign of abnormality.

A 69-year-old male patient with Machado-Joseph disease (MJD) presented with a mild cerebellar ataxia, global areflexia, and nystagmus. Magnetic resonance imaging showed cerebellar atrophy; brainstem atrophy, mainly pontine, and a linear abnormal bilateral hyperintense along the medial aspect of the globus pallidus internus on T2-weighted sequence and fluid-attenuated inversion recovery (FLAIR) ( Figure 1 ). This radiographic finding implies degeneration of the lenticular fasciculus. 1 The hyperintensity may be associated with degeneration of the subthalamic fascicles or the nigrostriatal dopaminergic fibers. 1 This finding is not pathognomonic of MJD, although it has been described in subjects with other types of spinocerebellar ataxias and in healthy elderly people. 2
